# A Hierarchical Core-Shell Structure of NiO@Cu_2_O-CF for Effective Non-Enzymatic Electrochemical Glucose Detection

**DOI:** 10.3390/nano15010047

**Published:** 2024-12-30

**Authors:** Yueyun Huang, Jiahua You, Yingru Ding, Yun Xie, Ting Wang, Fanglong Zhu, Weiping Gong, Zhenting Zhao

**Affiliations:** Guangdong Provincial Key Laboratory of Electronic Functional Materials and Devices, Huizhou University, Huizhou 516001, China; huangyy@hzu.edu.cn (Y.H.); 2207080116@stu.hzu.edu.cn (J.Y.); 2207080138@stu.hzu.edu.cn (Y.D.); xieyunxx@hzu.edu.cn (Y.X.); witton.wang@hzu.edu.cn (T.W.); flz_123@hzu.edu.cn (F.Z.); gwp@hzu.edu.cn (W.G.)

**Keywords:** NiO-coated Cu_2_O, non-enzymatic, electrochemical glucose detection

## Abstract

Non-enzymatic glucose detection is an effective strategy to control the blood glucose level of diabetic patients. A novel hierarchical core–shell structure of nickel hydroxide shell coated copper hydroxide core based on copper foam (Ni(OH)_2_@Cu(OH)_2_-CF) was fabricated and derived from NiO@Cu_2_O-CF for glucose sensing. Cyclic voltammetry and amperometry experiments have demonstrated the efficient electrochemical catalysis of glucose under alkaline conditions. The measurement displays that the fabricated sensor exhibits a detection scale of 0.005–4.5 mM with a detection sensitivity of 4.67 µA/µM/cm^2^. It has remarkable response/recovery times in respect of 750 μM glucose (1.0 s/3.5 s). Moreover, the NiO@Cu_2_O-CF shows significant selectivity, reliable reproducibility and long-term stability for glucose determination, suggesting it is a suitable candidate for further applications.

## 1. Introduction

Diabetes is a metabolic disease caused by the abnormal levels of glucose concentration in the human body. Nowadays, it is a serious chronic disease in the world. Diabetes is not only highly responsible for blindness, kidney failure, heart diseases, and strokes, but is also the leading cause of death [1,2,3,4]. “Silent killer” is another name for diabetes [5]. Diabetes was a direct cause of 1.5 million deaths in 2019 [6]. According to the World Health Organization (WHO), diabetes will become the seventh leading cause of death in the world by 2030 [3,7]. Considering the extremely large financial burden of diabetes and its serious complications, it is important to diagnosis diabetes early [6]. Monitoring glucose concentration is an effective technique in disease prevention and diagnosis.

Considerable efforts have been invested in the development of glucose determination, such as fluorescence [8,9,10], colorimetry [11,12], chemiluminescence [13,14], electrochemiluminescence [15] and the electrochemical technique [7,16,17,18]. Among these detecting methods, electrochemistry has been recognized as the promising approach for its inherent advantages such as convenience, rapid response, reliablity, outstanding sensitivity and the simplicity of operation. The electrochemical glucose sensors are generally divided into two major types, which are enzymatic and non-enzymatic methods [19]. Enzymatic glucose sensors, based on immobilizing glucolase on conductive substrates, possess high selectivity. However, the application of enzyme-based biosensors is limited by a lot of deficiencies, such as it being complicated to structure the electrodes, the low thermal and chemical stability of the enzyme, and it being expensive. Therefore, increasing studies focus on non-enzymatic glucose sensors [20,21,22,23,24,25,26,27,28,29,30]. Among a wide range of enzyme-free electrocatalytic materials, metal oxides, such as nickel oxide [31,32,33] and copper oxide [34,35,36,37,38], are suitable choices because they have the advantages of easy access and low cost.

To further enhance the electro-catalytic property of metal-oxide-based sensitive materials, preparing a three-dimensional multilayer structure is an effective approach [23,39,40]. In order to construct hierarchically sensitive materials, using copper foam (CF) as a substrate is an appropriate candidate. Furthermore, to increase the load of sensitive materials on a limited surface, designing a core-shell nano-structure is an excellent solution. Therefore, using nickel oxide and copper oxide to construct a core–shell structure on 3D copper foam and form a multicomponent hierarchical sensitive material could be a promising approach. It will have the advantages of a large specific surface area, efficient ion diffusion and electron transfer path.

This research drives the development of a flower spike-like structure composed of NiO and Cu_2_O, formed by the air annealing of Ni(OH)_2_ and Cu(OH)_2_ based on copper foam, labeled as NiO@Cu_2_O-CF. Within the newly created hierarchical architecture, Cu_2_O nanowires spontaneously formed on the CF and were subsequently encapsulated by NiO nanosheets. In the hierarchical core–shell structure design, the prevention of nickel oxide accumulation ensures optimal contact with glucose, thereby enhancing sensor efficiency. The electrocatalytic property of the constructed sensor was investigated by cyclic voltammetry and amperometry tests. The as-prepared sensor possesses excellent performance in terms of high sensitivity, effective selectivity, a fast response–recovery characteristic and a large liner range for glucose sensing. Therefore, the NiO@Cu_2_O-CF hierarchical structure is a promising material for sensitive glucose determination.

## 2. Materials and Methods

### 2.1. Materials and Reagents

Commercial Copper foams (CF) were purchased from Kunshan Luchuang ElectronicTechnology Co., Ltd. (Kunshan, China). All the reagents were obtained from Shanghai Aladdin Biochemical Technology Co., Ltd. (Shanghai, China) at analytical grade: ammonium persulfate ((NH_4_)_2_S_2_O_8_), sodium hydroxide (NaOH, 96%), concentrated hydrochloric acid (HCl), Nickel nitrate hexahydrate (Ni(NO_3_)_2_·6H_2_O, 97%), potassium chloride (KCl), sodium chloride (NaCl), fructose (Fru), sucrose (Suc), urea (UA, 99.0%), citric acid (CA) and sodium citrate (SSC, 98%).

### 2.2. Synthesis of NiO@Cu_2_O-CF

**Scheme 1:**
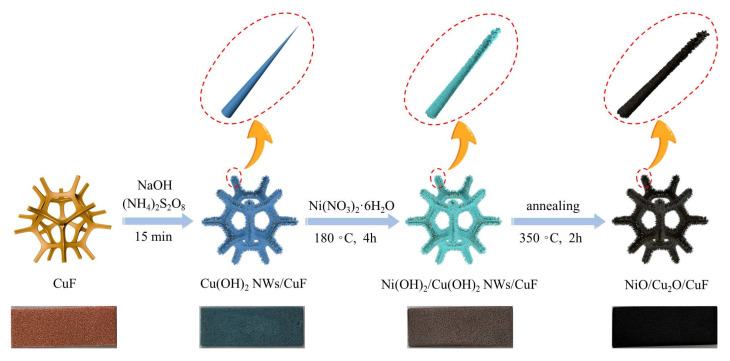
The illustration of the synthetic procedure and the photographs of the as-synthesized materials.

The synthetic procedure for NiO@Cu_2_O-CF is illustrated in Scheme 1 [25]. The illustration of the synthetic procedure and the photograph of the as-synthesized materials are shown in Scheme 1. Firstly, the copper foam substrate was soaked in 0.1 M HCl for 10 min, and then it was cleaned ultrasonically using DI water three times. Secondly, 16 mL 0.16 mol NaOH water solution and 44 mL 8 mmol (NH_4_)_2_S_2_O_8_ water solution were mixed and a colorless solution was obtained. Subsequently, CF was placed in the mixed solution for 15 min. Then, a obtained blue substrate was cleaned by absolute ethanol and DI water three times, which was marked as Cu(OH)_2_-CF.

The NiO@Cu_2_O-CF electrode was derived by Ni(OH)_2_@Cu(OH)_2_-CF annealing in air. Drawing on Sun’s study, the Ni(OH)_2_@Cu(OH)_2_-CF electrode was fabricated through a single-step solvothermal process [1]. As is typical, 40 mL methanol, containing 240 mg Ni(NO_3_)_2_·6H_2_O, was transferred into a 100 mL polytetrafluoroethylene autoclave. The as-synthesized Cu(OH)_2_-CF was placed into methanol solution. Meanwhile, the steel autoclave was maintained at 180 °C for 4 h and allowed to cool down naturally in atmospheric conditions. Then, the reacted foam was rinsed with DI water three times and dried in air. The obtained foam was named as Ni(OH)_2_@Cu(OH)_2_-CF. Finally, the obtained precursor, Ni(OH)_2_@Cu(OH)_2_-CF, was annealed at 350 °C with a heating rate of 5 °C/min for 2 h. After cooling down to room temperature, a black product was obtained, designated as NiO@Cu_2_O-CF. To prove the synthesis of NiO, we duplicated the methanol solution preparation and conducted the reaction under standard conditions, excluding the Cu(OH)_2_-CF. Subsequently, the produced powder was subjected to calcination at 350 °C for a duration of 2 h to yield the NiO powder.

### 2.3. Structural Characterization

The crystal structure of sensitive materials was characterized by X-ray diffraction (XRD) on a MiniFlex 600 X-ray diffractometer (600 W, Rigaku, Tokyo, Japan). The morphology of Ni(OH)_2_@Cu(OH)_2_-CF and NiO@Cu_2_O-CF were characterized by scanning an electron microscope (VEGA3, TESCAN, Bmo, Czech Republic). The hierarchical structure and chemical compositions of NiO@Cu_2_O-CF were analyzed by transmission electron microscopy (TEM) and energy dispersive spectroscopy (EDS), respectively. The distribution of the elements of NiO@Cu_2_O-CF was obtained by using the X-ray photoelectron spectroscopy (XPS, Thermo) spectrum. The specific surface area of Cu(OH)_2_-CF and NiO@Cu_2_O-CF were measured using the Tristar 3020 (Micromeritics instrument (Shanghai) Ltd., Norcross, GA, USA) physical adsorption instrument from Micromeritics.

### 2.4. Electrochemical Measurement

All the electrochemical experiments were completed using the electrochemical work station (HY-E1002A, Shenzhen Haoyang Technology Co., Ltd., Shenzhen, China). The Ag/AgCl (3 M KCl) electrode served as the reference, while the platinum electrode functioned as the counter electrode. The electrodes that were synthesized were employed as the working electrodes. Moreover, the electrochemical performances of obtained electrodes for glucose detection were evaluated by cyclic voltammetry and chronoamperometry tests. During the test cycle voltammetry, the scan rate was set at 50 mV/s, with the voltage sweep spanning 0 to 0.9 V.

## 3. Results and Discussion

### 3.1. Morphological and Structural Characterization

**Figure 1:**
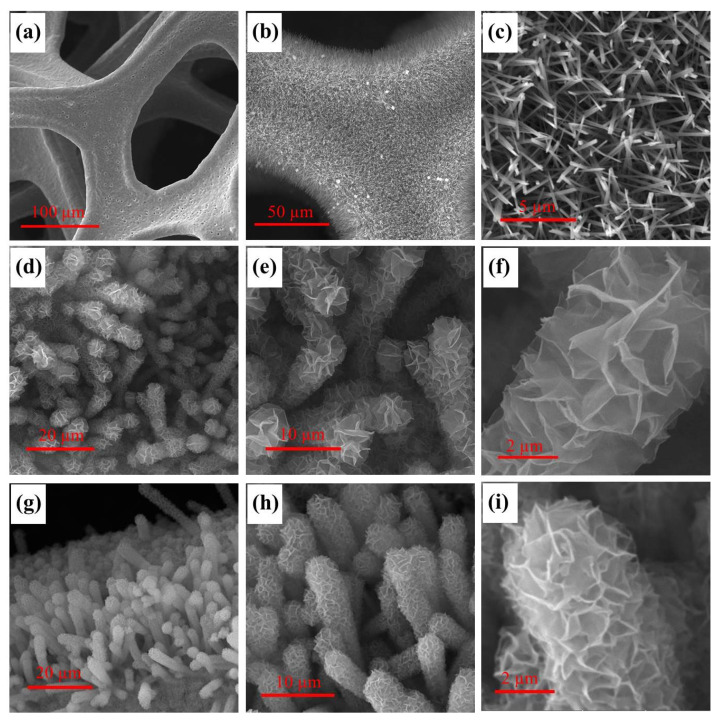
SEM image of CF (**a**), Cu(OH)_2_ nanowires (**b**,**c**), Ni(OH)_2_@Cu(OH)_2_-CF (**d**–**f**) and NiO@Cu_2_O-CF (**g**–**i**).

The SEM analysis was conducted to examine the morphologies of CF, Cu(OH)_2_-CF, Ni(OH)_2_@Cu(OH)_2_-CF and NiO@Cu_2_O-CF. The typical three-dimensional structure of CF can be observed in Figure 1a. Figure 1b,c depict the structure of Cu(OH)_2_-CF, featuring a uniform array of Cu(OH)_2_ nanowires that have colonized the copper foam’s surface. Clearly seen in Figure 1d–f, the Cu(OH)_2_ nanowires are enveloped by a hydrangea-like layer. The formed Ni(OH)_2_@Cu(OH)_2_-CF composite features a rod-like hierarchical architecture and a distinct hydrangea-like appearance. For the production of metal oxide-based sensory materials, the Ni(OH)_2_@Cu(OH)_2_-CF was subjected to air annealing, leading to the oxidation of nickel and copper hydroxides to NiO and Cu_2_O, respectively. As depicted in Figure 1g–i, the annealed NiO@Cu_2_O-CF maintained its morphology with minimal alteration, resembling its pre-annealed state.

**Figure 2:**
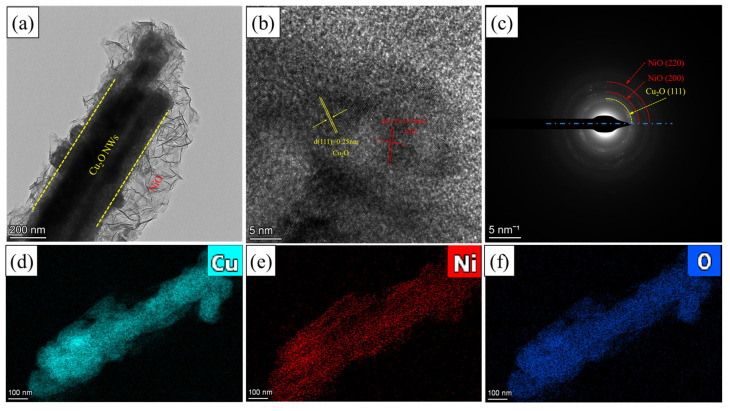
The TEM (**a**), HRTEM (**b**) and SAED (**c**) of NiO/Cu_2_O; The elemental mapping of Cu (**d**), Ni (**e**) and O (**f**).

Further visualization of the morphology and structural attributes of NiO@Cu_2_O-CF was achieved through TEM analysis and element mapping. The fragmented NiO@Cu_2_O, which was scraped from the surface of the CF substrate, was characterized by TEM as shown in Figure 2a. Clearly, the nanowire (diameter ≈ 350 nm) is surrounded by an ultrathin shell featuring a folded structure. The evident interface between the nanowire and shells indicates a typical hierarchical core–shell structure. Figure 2b displays the high-resolution TEM result of the core–shell structural NiO@Cu_2_O. The lattice space of 0.25 nm can correspond to the (111) plane of Cu_2_O, and the 0.20 nm was indexed as the (200) plane of NiO. In addition, the selected area electron diffraction (SAED) result in Figure 2c revealed that the direction rings can correspond to (200), (220) planes of NiO and (111) planes of Cu_2_O. Moreover, Figure 2d–f demonstrate the distribution of the Cu, Ni and O elements on NiO@Cu_2_O.

**Figure 3:**
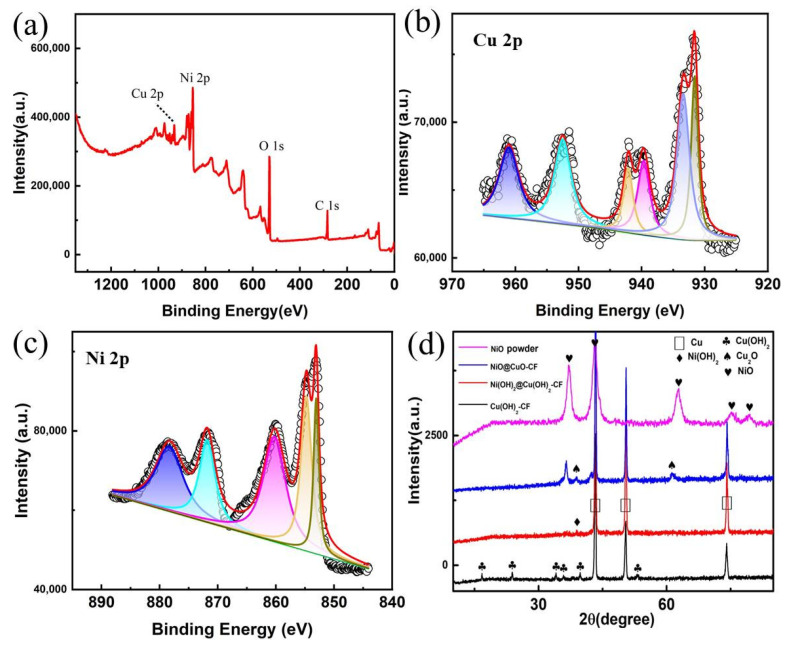
The chemical composition and crystalline structure analysis of NiO@Cu_2_O-CF: survey spectrum of XPS (**a**), Cu 2p spectrum of XPS (**b**), and Ni 2p spectrum of XPS (**c**); (**d**) the XRD patterns of as-synthesized materials.

Furthermore, the XPS technique was employed to analyze the chemical composition of NiO/Cu_2_O/CF. The complete spectrum in Figure 3a demonstrates the existence of Cu, Ni and O. The Cu 2p spectra in Figure 3b presents the fitted peaks with the binding energy of 931.6 eV, and 933.4 eV can be indexed as the Cu 2p3/2, evidencing the existence of Cu^+^. And the peaks of 952.5 eV are classified as the Cu 2p1/2. Figure 3c reveals the Ni 2p spectral region with Ni 2p 3/2 peaks located at 853 eV and 854.7 eV, while the peak at 871.8 eV is attributed to Ni 2p1/2, indicative of Ni^2+^ ions.

To further study the crystallographic structure of as-fabricated samples, the X-ray powder diffraction (XRD) pattern delineation of Cu(OH)_2_-CF, Ni(OH)_2_@Cu(OH)_2_-CF, NiO@Cu_2_O-CF and NiO powder were carried out and are shown in Figure 3d. It was found that the XRD pattern of as-synthesized materials agreed well with the standard PDF card of Cu (PDF#04-0836), Cu(OH)_2_ (PDF#013-0420), Ni(OH)_2_ (PDF#014-0117), Cu_2_O (PDF#05-0667) and NiO (PDF#47-1049), respectively. For NiO@Cu_2_O-CF, the NiO peak is faint. This may be due to the small amount of coverage on the nanowires. Consequently, an identical method was employed to synthesize the NiO powder. There are three peaks located at 36.5°, 42.4° and 61.6° which correspond to the (111), (200) and (220) crystal planes of Cu_2_O [41,42]. The diffraction peaks at 37.2°, 43.2°, 62.8°, 75.4° and 79.4° can be indexed to the (111), (200) (220), (311) and (222) crystal planes, respectively, of NiO [43]. The XRD data confirm the effective synthesis of the materials.

### 3.2. Electrochemical Properties of NiO@Cu_2_O-CF

**Figure 4:**
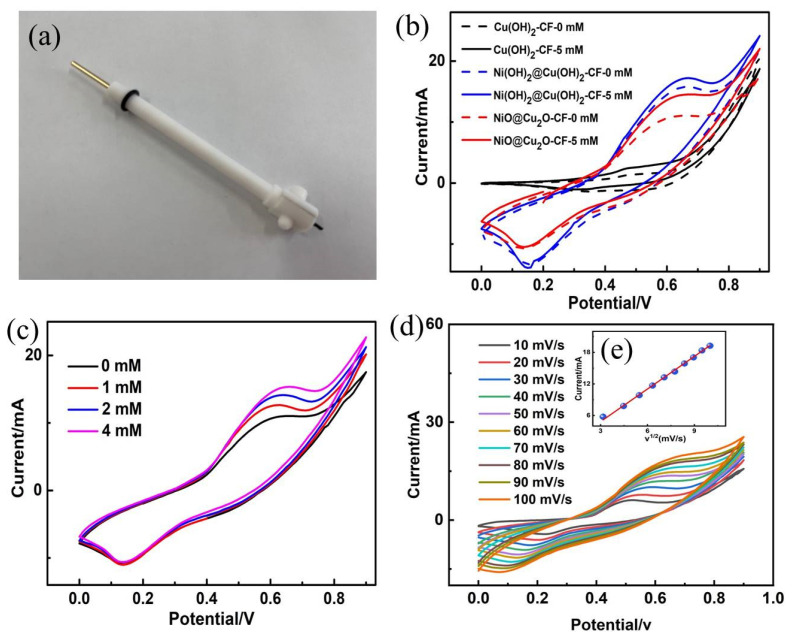
(**a**) The measurement setup of the working electrode; (**b**) CV curves (vs. Ag/AgCl) of Cu(OH)_2_-CF, Ni(OH)_2_@Cu(OH)_2_-CF and NiO@Cu_2_O-CF with 0 and 5.0 mM glucose in 0.1 M NaOH at 50 mV/s; (**c**) CVs (vs. Ag/AgCl) of NiO/Cu_2_O/CF with 0, 2.0 and 6.0 mM glucose; (**d**) the CV results that dependent on different scan rate (10–100 mV/s) in 0.1 M NaOH with 5.0 mM glucose; (**e**) inset image is the linear fitting of catalytic current increment vs. the square root of scan rate (ν^1/2^).

To facilitate testing, the synthesized sensitive material was trimmed into a 0.5 × 1.5 cm rectangle and subsequently secured with electrode clips for subsequent electrochemical experiments. And Figure 4a depicts the measurement setup of the working electrode. The electrochemical behavior of the synthesized electrodes was assessed using cyclic voltammetry. Figure 4b illustrates the CV results of as-fabricated electrodes, which were tested in 0.1 M NaOH with 5.0 mM and without glucose. For Cu(OH)_2_-CF, the oxidation current shows a minimal rise in the presence and absence of glucose. As the Cu(OH)_2_ nanowires were coated with Ni(OH)_2_ nanosheets, there was an enhancement in the electrocatalytic efficiency towards glucose, though the oxidation current observed a minimal rise, signifying that the Ni(OH)_2_ nanosheets have a limited catalytic capacity for glucose oxidation. When the as-fabricated Ni(OH)_2_@Cu(OH)_2_-CF was oxidized to NiO@Cu_2_O-CF, the CV exhibited obvious current increase once the glucose was added to the solution. Comparative experiments reveal that NiO/Cu_2_O exhibits superior electrochemical catalytic activity towards glucose compared to Cu(OH)_2_ and Ni(OH)_2_@Cu(OH)_2_. Additionally, as depicted in Figure 4c, there is a marked rise in the electrocatalytic current in response to increasing glucose concentrations, confirming that the NiO@Cu_2_O-CF electrode is sensitive to different glucose levels and is suitable for use as a glucose sensor.

The mechanism of electrochemically catalyzed glucose on NiO@Cu_2_O-CF is accompanied by the formation of CuOOH and NiOOH in NaOH solution and reacts with hydroxyl anions to form glucolactone [3,37,44]. In other words, the Ni^2+^ and Cu^+^ of NiO and Cu_2_O are oxidized to Ni^3+^ and Cu^3+^ in test solution. With the glucose added, glucose on the surface of NiO@Cu_2_O was oxidated via deprotonation. Meanwhile, the Ni^3+^ and Cu^3+^ was reduced to Ni^2+^ and Cu^2+^; the reactions can be expressed as follows [45]:

NiO + OH^−^ → NiOOH + e^−^ (1)

Cu_2_O + 2OH^−^ + H_2_O → 2Cu(OH)_2_ + 2e^−^ (2)

Cu(OH)_2_ + OH^−^ → CuOOH + H_2_O + e^−^ (3)

NiOOH + glucose → NiO + gluconolactone(4)

CuOOH + glucose → Cu(OH)_2_ + gluconolactone (5)

Furthermore, the electrochemical catalytic kinetics of NiO/Cu_2_O/CF were analyzed in 0.1 mM NaOH solution with 5.0 mM glucose under different scan rates (10–100 mV/s). As Figure 4c shows, the oxidation peak current increases gradually and presents a positive correlation with the scanning rate. Figure 4d,e displays a liner relationship between the anodic peak current and the square root of scan rates (ν^1/2^), which suggest the electrochemical reaction is a typical diffusion-controlled process.

### 3.3. Potentiostatic Catalytic Properties of NiO@Cu_2_O-CF

**Figure 5:**
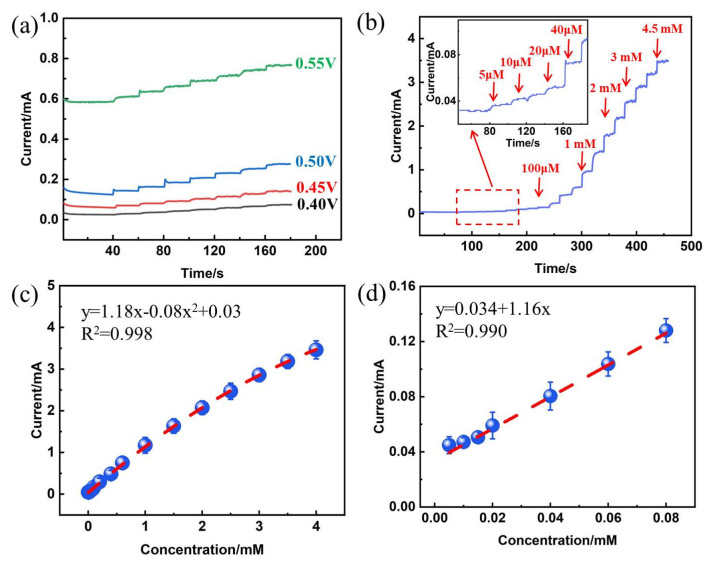
(**a**) Amperometric test curves of NiO@Cu_2_O-CF with continuous adding of glucose under different potentials: 0.40 V, 0.45 V, 0.50 V and 0.55 V; (**b**) amperometric titration experiment of NiO@Cu_2_O-CF at 0.45 V with 0.005–4.5 mM glucose, and the inset is the i–t curve in low glucose concentrations; (**c**) the quadratic polynomial fitting calibration line of the sensor; (**d**) the linear fitting calibration line of the sensor.

The amperometry technique was used to further estimate the electrochemical sensing characteristics of NiO@Cu_2_O-CF. Figure 5a shows the amperometric responses of NiO@Cu_2_O-CF at potential variations from 0.40 to 0.55 V in 0.1 M NaOH solution. When the potential was 0.40 V, the current response after adding glucose was very small and no obvious step shape was observed. Moreover, the background current will increase significantly as the applied potential increases. At the same times, it is considered that the higher the potential, the more interferences will be electrocatalyzed, resulting in a decrease in the selectivity. Therefore, 0.45 V is determined as the optimal potential. Subsequently, the typical i–t curve in Figure 5b was obtained by successively dropping the glucose solution to 50 mL 0.1 M NaOH with a 20 s interval at 0.45 V. According to the results, the NiO@Cu_2_O-CF presents a larger measurement scale for the glucose range from 0.005 mM to 4.5 mM. The inset image shows a low glucose concentration for the amperometric titration test range of 5 μM to 60 μM, which demonstrated that the electrode responded sensitively, even at a low glucose concentration. The quadratic polynomial fitting curve of catalytic current versus glucose concentration was defined as $y\;\left(mA\right)=0.00118x\;(\mu M)-{0.00008x}^{2}(\mu M)+0.000033$ (R^2^ = 0.9985) in Figure 5c. Additionally, Figure 5d displays the linear fitting curve between current and low glucose concentration with the equation: $y\;\left(mA\right)=0.00116x\;\left(\mu M\right)+0.000034$ (R^2^ = 0.9861). According to the fitting linear and geometric area of the electrode, the as-fabricated NiO@Cu_2_O-CF-based sensor possesses a sensitivity of 4.67 µA/µM/cm^2^.

**Figure 6:**
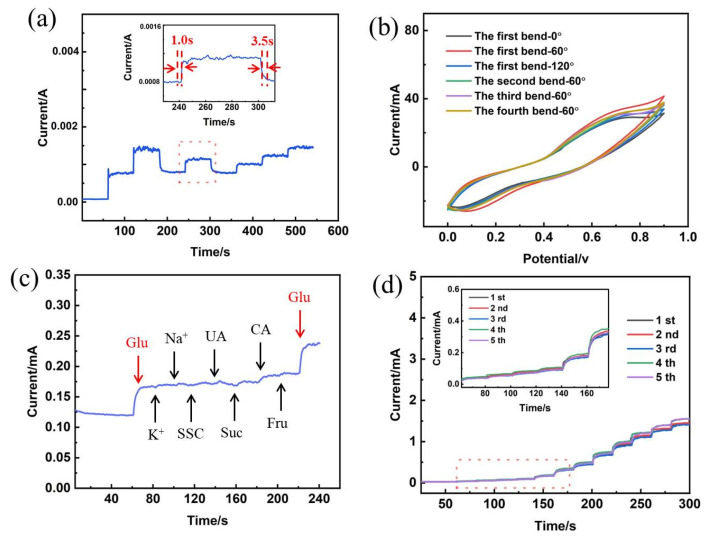
(**a**) The i–t test curve of NiO@Cu_2_O-CF when increasing and diluting the glucose concentration, and the inset image shows the response and recovery times toward 0.75 mM glucose; (**b**) the CVs of NiO@Cu_2_O-CF when it was bent to different angles (0°, 60° and 120°). (**c**) Interference test of NiO@Cu_2_O-CF when adding KCl, NaCl, sodium citrate, urea, sucrose, citric acid and fructose; (**d**) the recorded i–t test results of five sensors prepared by the same process and inset is the response current in low concentration.

Additionally, as well as sensitivity, the response–recovery behavior, anti-interference performance, reproducibility and stability also make a great difference in evaluating the electrochemical catalytic ability for an efficient sensor. Figure 6a recorded the response–recovery ability of NiO@Cu_2_O-CF when the glucose was increased and diluted. At first, the current increased sharply when the glucose solution was dropped into the electrochemical cell. Then, the glucose was diluted by adding NaOH solution and the catalytic current decreased. From the inset image of Figure 6a, it took about 1.0 s to reach a steady-state value in the reaction cell, and it recovered to the base line in about 3.5 s. This confirms that the NiO@Cu_2_O-CF possesses fast response and recovery characteristics in sensing glucose. In addition, the effect of the deformed electrode was detected by bending the electrode from 0° to 120°. According to the result shown in Figure 6b, there was no significant difference for the CV in the NaOH solution with 4 mM glucose, suggesting the good anti-bending performance of the as-constructed electrode.

**Figure 7:**
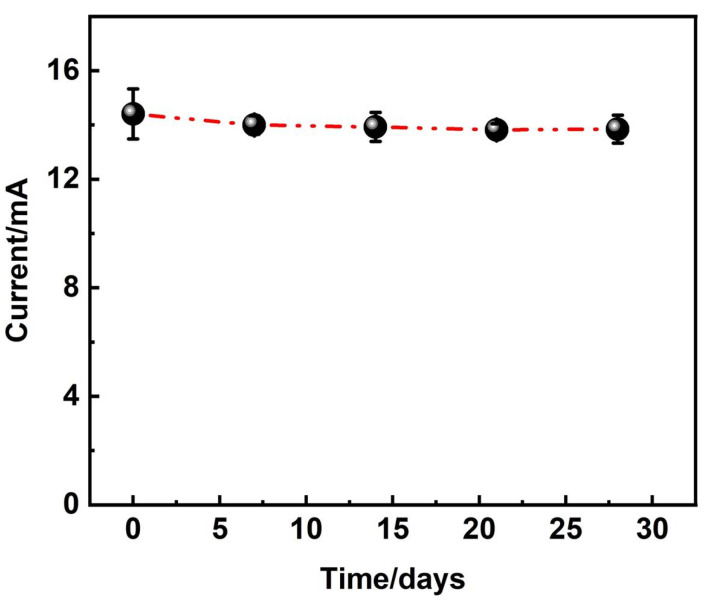
Long-term stability of NiO@Cu_2_O-CF electrode.

Selectivity is an essential ability for NiO@Cu_2_O-CF in practical application as an electrochemical sensor. As shown in Figure 6c, the interferences, such as KCl, NaCl, sodium citrate, urea, sucrose, citric acid and fructose, were added to the electrochemical cell in sequence. The concentration of the added interference was 100 µM. Compared to adding glucose, there was no significant current response with injecting those substances into the electrochemical cell. This indicates that the NiO@Cu_2_O-CF electrode possesses excellent selectivity for glucose determination. To evaluate the reproducibility of NiO@Cu_2_O-CF, we carried out an amperometry experiment on five electrodes fabricated by the same process. As shown by Figure 6d, almost all the electrodes respond with a similar current change with the glucose concentration range being 0.04–3.0 mM. The inset image is the results at a low glucose concentration range (0.04–0.4 mM). For evaluating the long-term stability of NiO@Cu_2_O-CF, cyclic voltammetry test was carried out on the same electrode in an electrochemical cell containing 5 mM glucose over 30 days (Figure 7). The relative standard deviation (RSD) of the current was calculated as 1.5% with 30 days, which demonstrated that the as-synthesized sensor presented outstanding long-term stability.

Finally, we compared this work with previous works which were developed using nickel oxide and copper oxide nanomaterials. We listed the key parameters in Table 1. We can conclude that the fabricated NiO@Cu_2_O-CF electrode exhibits good performance for glucose detection, including the high sensitivity and large linear range. Through the BET test, we found that the specific surface area of NiO@Cu_2_O-CF (4.26 m^2^/g) increased significantly compared with that of Cu(OH)_2_-CF (2.43 m^2^/g). Therefore, the possible reason is that the hierarchical core–shell structure can effectively improve the electrochemical performance for oxide nanomaterials.

## 4. Conclusions

In summary, we successfully fabricated a hierarchical core–shell structure named NiO@Cu_2_O-CF, in which Cu(OH)_2_ nanowires was encapsulated by Ni(OH)_2_ nanosheets on the Cu foam substrate. Then, we smartly treated Ni(OH)_2_@Cu(OH)_2_-CF at 350 °C to obtain NiO@Cu_2_O-CF with the same framework. The electrochemical detections demonstrated that the NiO@Cu_2_O-CF electrode presented the property of excellent glucose sensing, including high sensitivity (1.49 µA/µM/cm^2^), a large linear range (0.005–4.5 mM) and fast response/recovery times (1.0 s/3.5 s). In addition, the NiO@Cu_2_O-CF electrode also exhibited good reproducibility, selectivity, anti-folding performance and long-term stability. Therefore, the as-synthesized electrode has great potential and prospects to work as an enzyme-free glucose sensor.

## Figures and Tables

**Table 1:** A performance summary of nickel oxide and copper oxide-based nonenzymatic glucose sensors.

Materials	Sensitivity (μA/µM/cm^2^)	Linear Range (μM)	Ref.
N-CuO/Cu_2_O:NiO film	1.13	0.05–2.74	[46]
g-C_3_N_4_/NiO/CuO	0.362	0.0004–8.5	[47]
NiO/CuO	112.08 μA/mM	0.14–180.00 mM	[48]
CuO/NiO@GCE	0.0397	0.04–4.86	[49]
CuO/NiO-0.4NFs	1.32	0.01–10.0	[50]
Ni-CuO/Cu/Cu_2_O	0.208	0.01–8.0	[51]
NiO@Cu_2_O-CF	4.67	0.005–4.5	This work

## Data Availability

Data are contained within the article.
